# Acute mental health presentations before and during the COVID-19 pandemic

**DOI:** 10.1192/bjo.2021.970

**Published:** 2021-07-16

**Authors:** Naaheed Mukadam, Andrew Sommerlad, Jessica Wright, Abigail Smith, Aleksandra Szczap, Solomis Solomou, Rohan Bhome, Roshan Thayalan, Esha Abrol, Golnar Aref-Adib, Lucy Maconick, Dominic Aubrey-Jones, Senem Tugrul, Melanie Knowles, Helen Menys, Shivanthi Sathanandan, Sarah Moslehi, Jonathan Huntley, Kathy Liu, Juan Carlos Bazo-Alvarez

**Affiliations:** Division of Psychiatry, University College London, UK; and Camden and Islington NHS Foundation Trust, UK; Division of Psychiatry, University College London, UK; and Camden and Islington NHS Foundation Trust, UK; Camden and Islington NHS Foundation Trust, UK; Camden and Islington NHS Foundation Trust, UK; Camden and Islington NHS Foundation Trust, UK; Camden and Islington NHS Foundation Trust, UK; Camden and Islington NHS Foundation Trust, UK; Camden and Islington NHS Foundation Trust, UK; Camden and Islington NHS Foundation Trust, UK; Division of Psychiatry, University College London, UK; and Camden and Islington NHS Foundation Trust, UK; Division of Psychiatry, University College London, UK; and Camden and Islington NHS Foundation Trust, UK; Camden and Islington NHS Foundation Trust, UK; Camden and Islington NHS Foundation Trust, UK; Camden and Islington NHS Foundation Trust, UK; Camden and Islington NHS Foundation Trust, UK; Camden and Islington NHS Foundation Trust, UK; Camden and Islington NHS Foundation Trust, UK; Division of Psychiatry, University College London, UK; and Camden and Islington NHS Foundation Trust, UK; Division of Psychiatry, University College London, UK; and Camden and Islington NHS Foundation Trust, UK; Department of Primary Care and Population Health, University College London, UK; and School of Medicine, César Vallejo University, Peru

**Keywords:** Suicide, community mental health teams, self-harm, depressive disorders

## Abstract

**Background:**

A number of community based surveys have identified an increase in psychological symptoms and distress but there has been no examination of symptoms at the more severe end of the mental health spectrum.

**Aims:**

We aimed to analyse numbers and types of psychiatric presentations to inform planning for future demand on mental health services in light of the COVID-19 pandemic.

**Method:**

We analysed electronic data between January and April 2020 for 2534 patients referred to acute psychiatric services, and tested for differences in patient demographics, symptom severity and use of the Mental Health Act 1983 (MHA), before and after lockdown. We used interrupted time-series analyses to compare trends in emergency department and psychiatric presentations until December 2020.

**Results:**

There were 22% fewer psychiatric presentations the first week and 48% fewer emergency department presentations in the first month after lockdown initiated. A higher proportion of patients were detained under the MHA (22.2 *v*. 16.1%) and Mental Capacity Act 2005 (2.2 *v*. 1.1%) (*χ*^2^(2) = 16.3, *P* < 0.0001), and they experienced a longer duration of symptoms before seeking help from mental health services (*χ*^2^(3) = 18.6, *P* < 0.0001). A higher proportion of patients presented with psychotic symptoms (23.3 *v*. 17.0%) or delirium (7.0 *v*. 3.6%), and fewer had self-harm behaviour (43.8 *v*. 52.0%, *χ*^2^(7) = 28.7, *P* < 0.0001). A higher proportion were admitted to psychiatric in-patient units (22.2 *v*. 18.3%) (*χ*^2^(6) = 42.8, *P* < 0.0001) after lockdown.

**Conclusions:**

UK lockdown resulted in fewer psychiatric presentations, but those who presented were more likely to have severe symptoms, be detained under the MHA and be admitted to hospital. Psychiatric services should ensure provision of care for these patients as well as planning for those affected by future COVID-19 waves.

## The Coronavirus pandemic

The novel Coronavirus was first identified in patients with viral pneumonia in Hubei province, China, in December 2019.^1^ The virus rapidly spread around the world, resulting in the World Health Organization declaring a public health emergency of international concern on 30 January 2020 and a pandemic on 11 March 2020. Because of its highly infectious nature, many countries initiated physical distancing measures and closures of non-essential businesses and services. This lockdown was announced in the UK on 23 March 2020. The potential impact of COVID-19 on mental health has been a focus of discussion since the start of the pandemic, and particularly since the introduction of physical distancing measures.

Previous pandemics have been associated with an increase in neuropsychiatric symptoms such as confusion, anxiety and depression,^2,3^ and living through a pandemic has the potential to increase stress, anxiety, depression and psychological distress.^4,5^ A review of neuropsychiatric symptoms related to COVID-19 has found that delirium and agitation are relatively common symptoms in intensive care units. There are also serious concerns about the psychological effects of physical distancing and isolation, with recognition of their potential to both cause new symptoms in those with no prior mental illness and to worsen mental states of those with pre-existing psychiatric disorders.^6,7^

## Impact on psychiatric symptoms

A number of community-based surveys have identified an increase in psychological symptoms and distress,^8–12^ but there has been no examination of symptoms at the more severe end of the mental health spectrum. Only one study has examined trends in acute psychiatric presentations before and during the pandemic, finding that presentations to one German mental health emergency service were 38% lower during the week after the introduction of partial lockdown compared with the equivalent week during 2019.^13^ This information is important for mental health service planning in advance of future waves of COVID-19 in the UK and other countries.

In this paper, we therefore aimed to describe the number and types of emergency psychiatric presentations to five acute mental health assessment centres before and at the peak of COVID-19 in London; analyse changes in trends of presentations to accident and emergency (A&E) departments covering the same geographical areas; and compare with data from the equivalent months of the previous year, to inform planning for future demand on mental health services and practice and policy for future waves of this pandemic.

## Method

### Setting and participants

We obtained service-level data about numbers of presentations to three acute mental health liaison (consultation psychiatry) teams and two acute mental healthcare centres in central London between 1 January 2019 and 31 December 2020. In addition, we obtained detailed electronic clinical case records for all presentations to these services between 1 January 2020 and 30 April 2020. We defined acute services as any specialist service where those with psychiatric symptoms can seek urgent assessment and care. Mental health liaison services provide mental health assessment and treatment for people presenting to A&E departments in acute hospitals and for people requiring mental health input as in-patients in acute hospitals. One of the acute mental healthcare centres was a ‘place of safety’, which was established, unrelated to the COVID-19 pandemic, in January 2020. It was a designated place to assess patients who were placed under temporary section of the Mental Health Act 1983 by police for their safety or the safety of others. The other mental healthcare centre was opened in response to the COVID-19 pandemic on 23 March 2020 to divert patients with primary mental health presentations from the three mental health liaison teams at the local acute hospitals’ A&E departments. Our assessment of psychiatric presentations and comparisons with A&E department were therefore partly to establish the impact of this restructuring of our acute psychiatric services.

### Data extraction

One of 17 research team members (N.M., A. Sommerlad, J.W., A. Smith, A. Szczap, S. Solomou, R.B., R.T., E.A., G.A.-A., L.M., D.A.-J., S.T., S.M., M.K., H.M. and S. Sathanandan), who are all psychiatrists with between 1 and 15 years of experience in practicing clinical psychiatry, reviewed the notes for each patient's clinical episode and extracted information on a standardised data extraction form that the authors designed.

Recorded information about demographic characteristics was age at referral, gender, ethnicity (White British, White other, Black/Black British, East Asian, South Asian, other, not recorded), whether the patient was known to mental health services, and the presence of a previous primary mental health diagnosis (following a hierarchy of mental health diagnoses whereby the first of the following diagnoses trumped subsequent comorbid diagnoses^14^: dementia, substance misuse, psychotic disorder, bipolar affective disorder, affective disorder, anxiety disorder, personality disorder, intellectual disability, other, none).

Information recorded about the index clinical episode was referral date, legal status on presentation (Mental Health Act section,^15^ Mental Capacity Act 2005,^16^ informal), primary presenting complaint (intoxication, delirium/confusion, self-harm/suicidal ideation or action, violence or aggression to others, psychotic symptoms, depressive symptoms, anxiety symptoms, other), duration of these symptoms (<1 day, 1–3 days, 4 days to 1 week, >1 week), outcome of initial assessment (ongoing mental health liaison team follow-up for patients admitted to acute general hospital, discharge with no mental health follow-up, home treatment team, other community mental health team, referral to memory service, psychiatric in-patient admission, other service), source of referral, and dates and duration of follow-up by psychiatry team. COVID-19 status was also recorded (positive antigen test, suspected, negative antigen test, not known or suspected).

We obtained information about the number of monthly A&E department attendances between 1 January 2019 and 31 December 2020 from the NHS England website (https://www.england.nhs.uk/statistics/statistical-work-areas/ae-waiting-times-and-activity/) at the three acute hospitals where our acute mental health liaison teams were situated. These three hospitals account for around 11% of London and 2% of all England A&E department presentations.

We used the National Institute for Health Research Health Research Authority online tool, which provided an exemption for the need for ethical review as this project is classified as a service evaluation (see Supplementary Appendix 1 available at https://doi.org/10.1192/bjo.2021.970) and we did not seek consent from participants as we retrospectively reviewed their notes and no patients are identifiable from information presented in the study.

### Statistical analyses

We used Stata version 16 for Windows (StataCorp 2019) for all analyses and Microsoft Excel for Windows for graphs. We calculated total mental health attendances to acute services (per week and per month) and A&E department attendances in the same hospitals per month from January 2019 to December 2020. We calculated the percentage of A&E department attendances that were referred to mental health liaison services each month. As a significant proportion of patients would have been diverted to the newly opened acute mental health centre during the pandemic, we combined acute psychiatric presentations across all settings for this calculation.

We used an interrupted time-series (ITS) analysis to compare trends in mental health presentations per week before and after lockdown. This is a widely accepted method for comparing data before and after public health interventions.^17,18^ ITS controls for time-independent confounders by design, so any change in the after-period trends can be attributed to lockdown, assuming there is no other time-variant confounders operating.^19^ We hypothesised that there would be an abrupt change in presentations after lockdown and that the longer-term trends would also be different before and after lockdown. For modelling the ITS, we fitted Poisson regression models with linear splines (i.e. one knot at the lockdown time point). Consistent with the hypothesis, this model included a variable for the change in intercepts (i.e. abrupt change) and two different slopes (i.e. before and after lockdown trends). We adjusted this model for overdispersion by including a Pearson *χ*^2^-based dispersion parameter. Autocorrelation and seasonality were controlled by adding Fourier terms to the model. We performed a sensitivity analysis to see if another feasible impact model (i.e. gradual instead of abrupt change) fit the data better (details are available in Supplementary Appendix 1). A similar ITS model was fitted for A&E department presentations, but using monthly data.

For the data extracted from patient electronic notes, we compared the data for the periods before and after lockdown, using the *t*-test to compare means for continuous variables and the *χ*^2^-test to compare percentages for categorical variables.

## Results

### Number and trends of acute psychiatric and non-psychiatric A&E department presentations

**Fig. 1:**
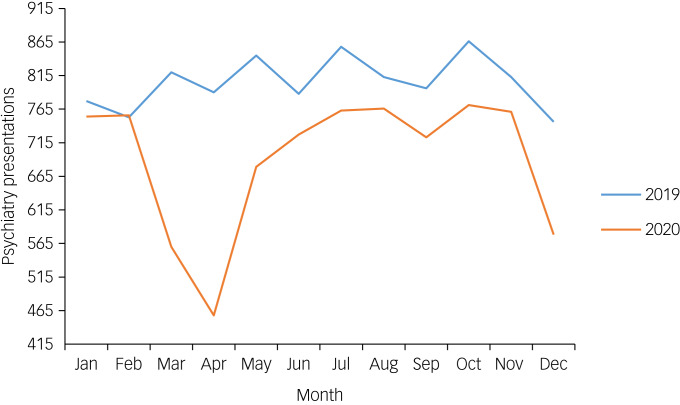
Psychiatric presentations per month, from January 2019 to December 2020.

**Fig. 2:**
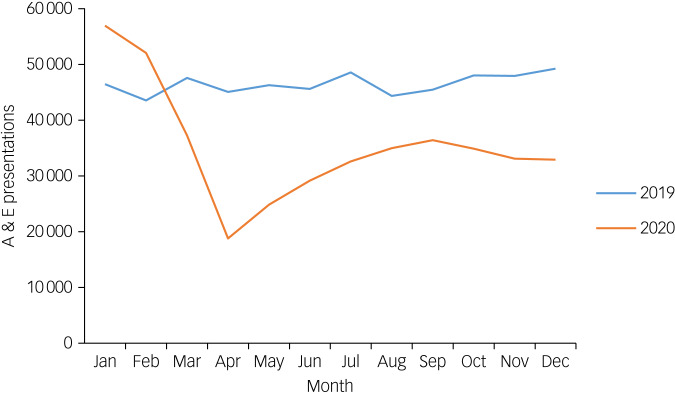
Accident and emergency department (A&E) presentations per month, from January 2019 to December 2020.

A&E department attendances were, on average, 46 497 per month from January to December 2019 (total 557 958), and 35 328 per month for the same time period in 2020 (total 423 930). Mental health attendances were, on average, 805 per month from January to December 2019 (total 9665) and 691 per month from January to December 2020 (total 8296). On average, around 2% of A&E department presentations were referred to mental health liaison teams per month throughout the time period surveyed. Figures 1 and 2 show presentations per month for 2019 and 2020 for acute psychiatric presentations and A&E department presentations, respectively. They both show that there was a substantial decline in both A&E department and psychiatric presentations from March 2020 onward, which did not recover to pre-pandemic levels.

**Fig. 3:**
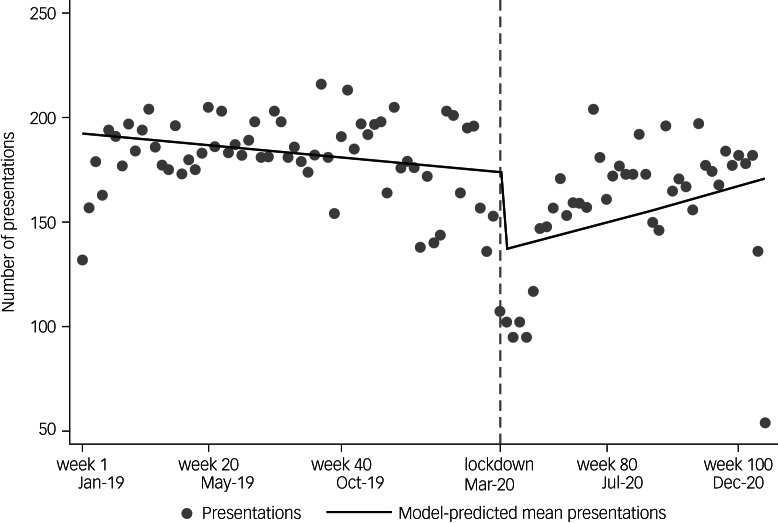
Interrupted time-series analysis showing weekly number of psychiatric presentations between 1 January 2019 and 31 December 2020, and abrupt and long-term change in weekly presentations (solid line) after 23 March 2020 (‘lockdown’; dashed line).

The ITS analysis showed abrupt reduction of psychiatric presentations after the lockdown: 1 week after the lockdown initiation on 23 March 2020, the average number of presentations was 78.4% (95% CI 67.4–91.2, *P* = 0.002) of the pre-lockdown average, dropping from approximately 175 to 140 presentations in the first week after lockdown (Table 1 and Fig. 3). The long-term slope before lockdown showed a negligible rate of decrease in psychiatric presentations (−0.2% per week, 95% CI −0.4 to 0.04, *P* = 0.123). This long-term trend changed in the weeks after lockdown (+1% per week, 95% CI 0.5–1.1, *P* = 0.003). In the sensitivity analysis, this model performed better than the alternative impact model of gradual change after lockdown (see Supplementary Appendix 1, section 3). We attempted an ITS with monthly referral data for psychiatry, but this model was weaker because of the smaller numbers of time points (see Supplementary Appendix 1, section 5).

**Table 1:** Change in the number of presentations before and after lockdown, estimated by interrupted time-series models

Relative change^a^	Psychiatric presentations (change on a weekly basis)						A&E department presentations (change on a monthly basis)					
	Unadjusted^b^			Adjusted^c^			Unadjusted^b^			Adjusted^c^		
	Relative change	95% CI	*P-*value	Relative change	95% CI	*P-*value	Relative change	95% CI	*P-*value	Relative change	95% CI	*P-*value
Immediate change in number of presentations at lockdown	0.8077	(0.7156–0.9116)	0.001	0.7844	(0.6746–0.9121)	0.002	0.4863	(0.3953–0.5980)	<0.001	0.5213	(0.4049–0.6712)	<0.001
Change in number of presentations after lockdown	1.0060	(1.0019–1.0102)	0.004	1.0057	(1.0004–1.0110)	0.033	1.0538	(1.0215–1.0870)	0.001	1.0415	(1.0025–1.0820)	0.037
Change in number of presentations before lockdown	0.9986	(0.9967–1.0005)	0.157	0.9984	(0.9964–1.0004)	0.123	1.0044	(0.9928–1.0160)	0.461	1.0025	(0.9904–1.0147)	0.686

The total number of time points for the interrupted time-series of psychiatric representations is 104 weeks. The total number of time points for the interrupted time-series of A&E department presentations is 24 months. A&E, accident and emergency.

a. Relative change refers to the number of presentations in a given week/month divided by the number of presentations in the previous week/month. For example, a relative change of 0.78 represents that the number of presentations in the week when the lockdown started was, on average, 78% of the number of presentations in the previous week. In other words, there were an average reduction of 22% in the number of psychiatric presentations that occurred in the immediate week after lockdown.

b. Adjusted for overdispersion only.

c. Adjusted for overdispersion, autocorrelation and seasonality.

**Fig. 4:**
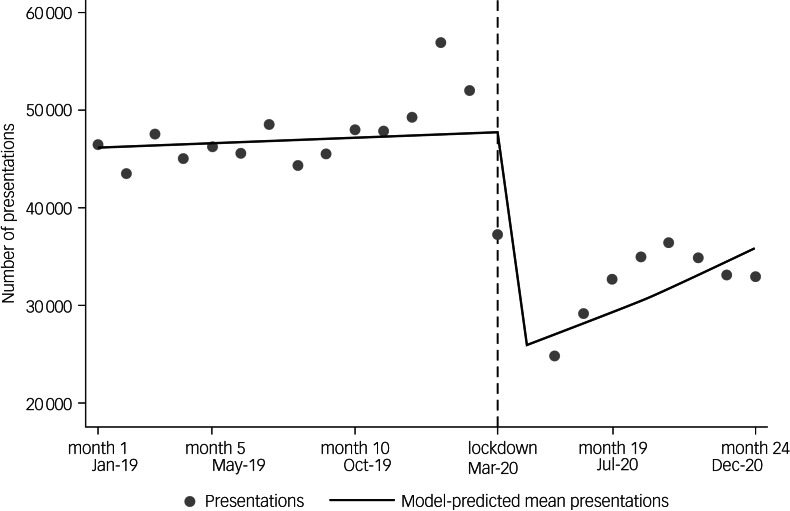
Interrupted time-series analysis showing monthly number of accident and emergency department presentations between 1 January 2019 and 31 December 2020, and abrupt and long-term change in monthly presentations (solid line) after 23 March 2020 (‘lockdown’; dashed line).

The second ITS analysis showed the reduction of A&E department presentations after the lockdown was also abrupt: 1 month after the lockdown started, the average number of presentations was 52.1% (95% CI 40.5–67.1, *P* ≤ 0.001) of the pre-lockdown average (Table 1 and Fig. 4). The long-term slope before lockdown showed a slight rate of decrease in A&E department presentations (0.2% per month, 95% CI −1.0 to 1.5, *P* = 0.686). Following the immediate decline after lockdown, the monthly rate of presentations then increased in the months post-lockdown, but remained below pre-lockdown levels (+4.1% per month, 95% CI 0.3–8.2, *P* = 0.37) (see Supplementary Appendix 1, section 5).

### Characteristics of patients presenting to psychiatric services before and after lockdown, between January 1 and April 30 2020

There were 2534 psychiatric presentations in total from 1 January to 30 April 2020, of whom 1988 presented before lockdown (1 January to 23 March) and 546 presented after lockdown (24 March to 30 April). Prior to lockdown, COVID-19 status for 99% of patients was unknown and only 0.1% tested positive. After lockdown, 71% had unknown COVID status, 9% were positive, 12% were negative and 8% were suspected.

Table 2 shows demographics of people presenting to mental health services before and after lockdown. Detailed clinical records review for January to April 2020 indicated that mean age for those presenting after 23 March 2020 (lockdown) was around 3 years more than before lockdown (95% CI 4.3–1.1, *P* = 0.0013). After lockdown, the proportion of men versus women increased (from 47.2 to 52.2%, *P* = 0.038), compared with the period before lockdown. There were no significant differences in ethnic group percentages before and after lockdown but the proportion of people from out of the local area decreased significantly (from 54.7 to 45.2%, *P* < 0.0001).

**Table 2:** Demographics of people presenting to acute mental health services before and after lockdown (from January to April 2020)

Characteristic	Before lockdown (*n* = 1988)	After lockdown (*n* = 546)	*t*-test/*χ*^2^(d.f.)	*P*-value
Age, years	41.1 (17.0)	43.8 (18.0)	*t* = −3.2	*P* = 0.0013
			d.f. = 2532	
Gender			*χ*^2^(1) = 4.3	*P* = 0.038
Female	52.8%	47.8%		
Male	47.2%	52.2%		
Ethnicity			*χ*^2^(5) = 7.4	*P* = 0.194
White British	44.1%	49.2%		
White Other	21.2%	19.1%		
Black/Black British	13.4%	14.9%		
South Asian	6.9%	5.6%		
East Asian	2.7%	1.7%		
Local/out of area			*χ*^2^ = 15.3	*P* < 0.0001
Local	45.3%	54.7%		
Out of area	54.8%	45.2%		

Numbers are mean (s.d.) for numerical variables and percentages for categorical variables.

Table 3 compares people presenting to mental health services with regards to the clinical characteristics of their psychiatric presentation before and after lockdown. There was an increase in proportion of people who were not known to mental health services previously, but this was not statistically significant. There was an increase in proportion of people presenting with existing diagnoses of psychotic disorders, personality disorders, bipolar affective disorder and dementia, but there was a lower proportion with previous diagnoses of anxiety disorders and substance misuse disorders (*χ*^2^(8) = 51.8, *P* < 0.0001).

**Table 3:** Characteristics of psychiatric presentations before and after lockdown

Characteristic	Before lockdown (*n* = 1988)	After lockdown (*n* = 546)	*t*-test/*χ*^2^	*P*-value
Known to mental health services previously			*χ*^2^(2) = 5.3	*P* = 0.071
Yes	66.5%	65.8%		
No	30.9%	33.2%		
Unclear	2.7%	1.1%		
Primary pre-existing mental health diagnosis			*χ*^2^(8) = 51.8	*P* < 0.0001
None	22.8%	23.4%		
Personality disorder	21.1%	24.9%		
Affective disorder	18.1%	7.7%		
Psychotic disorder	17.2%	24.2%		
Substance misuse	9.0%	7.9%		
BPAD	5.0%	5.7%		
Anxiety disorder	3.6%	1.7%		
Dementia	1.8%	2.0%		
Intellectual disability	1.6%	2.6%		
Legal status			*χ*^2^(2) = 16.3	*P* < 0.0001
Informal	82.9%	75.6%		
MHA	16.1%	22.2%		
DOLS	1.1%	2.2%		
Presenting complaint			*χ*^2^(7) = 28.7	*P* < 0.0001
Suicide/self-harm^a^	52.0%	43.8%		
Psychotic symptoms	17.0%	23.3%		
Depressive symptoms	6.7%	6.0%		
Violence to others	4.0%	5.0%		
Anxiety	4.3%	4.0%		
Intoxication	3.3%	3.3%		
Delirium	3.6%	7.0%		
Other	9.1%	7.7%		
Duration of presenting symptoms before help-seeking			*χ*^2^(3) = 18.6	*P* < 0.0001
<1 day	51.2%	42.1%		
1–3 days	14.0%	19.6%		
4–7 days	5.6%	7.4%		
>1 week	29.1%	30.9%		
Duration of referral episode (days)	1.9 (0.1)	2.2 (0.2)	*t* = −0.3,	*P* = 0.25
			d.f. = 2532
Outcome of assessment			*χ*^2^(6) = 42.8	*P* < 0.0001
Discharge (no follow-up)	28.8%	28.0%		
In-patient admission	18.3%	22.2%		
CMHT	13.2%	14.1%		
HTT	17.4%	15.0%		
MHLT follow-up	9.0%	15.0%		
Memory service	0.6%	0.6%		
Other service	12.7%	5.1%		

Numbers are mean (s.d.) for numerical variables and percentages for categorical variables. BPAD, bipolar affective disorder; MHA, Mental Health Act; DOLS, Deprivation of Liberty Safeguards: CMHT, community mental health team; HTT, home treatment team; MHLT, mental health liaison team.

a. Thoughts or actions.

A higher proportion of people presented with psychotic symptoms, violence to others and delirium, but the proportion presenting with self-harm or suicidal acts/thoughts and with depression or anxiety symptoms was lower (*χ*^2^(7) = 28.7, *P* < 0.0001). Absolute numbers with delirium were similar (around six per week), but the numbers of people presenting with self-harm thoughts and acts and those with depression and anxiety more than halved (see Supplementary Appendix 1, section 7). Only five patients were recorded as specifically mentioning concern about COVID-19 itself; one was documented as having a delusion relating to coronavirus and another was bereaved by COVID-19.

A smaller proportion of patients presented voluntarily (75.6 *v*. 82.9%) and higher proportions of patients were detained under the Mental Health Act (22.2 *v*. 16.1%) and Mental Capacity Act (2.2 *v*. 1.1%) (*χ*^2^(2) = 16.3, *P* < 0.0001) after lockdown. There was in increase in how long people had symptoms before seeking help from mental health services (*χ*^2^(3) = 18.6, *P* < 0.0001). More people were admitted to psychiatric in-patient units after assessment after lockdown compared with before (2.2 *v*. 18.3%) and fewer were discharged with no mental health follow-up (*χ*^2^(6) = 42.8, *P* < 0.0001). There was no difference in the length of assessment and treatment by acute services before and after lockdown (mean difference −0.3 days, 95% CI −0.8 to 0.2, *P* = 0.25).

## Discussion

Our study provides detailed analyses of acute psychiatric presentations before and during the COVID-19 pandemic, and considers trends before and after the period of lockdown in England, as well as comparisons with A&E services. Psychiatric presentations reduced by around 22% the first week immediately following lockdown. Monthly A&E department presentations declined by 48% the first month after lockdown. We cannot directly compare these because of the different time units, and an ITS for monthly psychiatric presentations was not appropriate.

We found an increase in the use of mental health legislation and more in-patient admissions after lockdown. We additionally showed an increase in proportion of presentations with psychosis, delirium and violence, but a decrease in self-harm and suicidal ideation and acts. Changes in proportions of most presentations was mostly driven by a decrease in numbers presenting with self-harm thoughts and acts rather than an increase in numbers presenting with delirium and violence, as these remained similar. Although we did conduct multiple tests, these were pre-planned and significant results had a *P-*value of <0.0001 in most cases, which is below the *P*-value necessary (*P* = 0.0045 for 11 tests) to show statistical significance if we apply a Bonferroni correction for multiple tests.^20^

Our results were similar to those found in Germany, where A&E department presentations declined by almost 27%, but affective disorder presentations declined by 42% and organic mental disorder presentations declined by >50%.^13^ Other studies conducted in the UK that used ITS have also shown a sharp decline in referrals to liaison psychiatry services, with one finding a reduction of around 40% in referrals after lockdown^21^ and another not quantifying the decrease.^22^ Our finding of a smaller reduction in referrals could be because of restructuring of local psychiatric services to divert people away from A&E departments, which may be an argument for considering their use in future waves of the pandemic. Other studies also showed a recovery period after the initial lockdown, similar to our findings,^23^ but neither conducted longer-term follow-up to the end of December as we did, so our finding of no increase in referrals beyond previous baseline is potentially reassuring and could indicate mental health needs being met in other services.

The decrease in suicidal and self-harm ideation and acts corroborates previous studies showing a decrease in suicidality during and after major disasters,^24,25^ and could be because of a greater sense of belonging and reduction in interpersonal risk factors.^26^ However, it could also be a result of people with self-harm thoughts or actions not seeking help because of concern for their safety in hospital settings, or lacking the usual support networks to encourage them to seek help. Fewer people with depressive and anxiety symptoms presented to acute psychiatric services after lockdown, representing a smaller proportion of the acute mental health case-load. This could reflect a greater threshold for seeking help or may be attributable to increased provision in the community for these symptoms, especially for those with established diagnoses.

### Implications

This study provides evidence not only of reduced presentations to acute mental health services, but also of greater severity of symptoms, greater delay in help-seeking, increased use of mental health legislation and increased likelihood of the need for admission and psychiatric follow-up after compared with before lockdown. Mental health services were restructured to divert people away from acute hospitals and to ensure mental health needs of the community are met, but despite this, there is an increase in severity of mental health conditions, which may place extra demand on in-patient units and community mental health services.

In addition, the reduction in number of patients assessed by these teams implies a large number of people not seeking help from services because of the lockdown, which in turn suggests a potential future surge in demand. Based on our modelling, a drop of 22% means around 35–40 people in the first week after lockdown were not seeking help from these acute services when they previously may have done. Some of them may have received help from elsewhere, but others may not have done so, risking symptoms worsening over time, potentially leading to a rebound increase in presentations in future. Although many of the people presenting to acute services were discharged with no formal follow-up, they will all have undergone assessment by a trained mental health professional, including receiving advice, signposting and contact details for relevant services. Those people who did not present to acute mental health services during this time may have had support or telephone contact with their general practitioner or community service but our study could not survey this group to assess whether this occurred, and if so, whether it had an effect on their symptoms.

### Strengths and limitations

We were able to assess the effects on numbers of people seeking help for mental health crises in five different centres. In addition, our detailed clinician-led review of clinical records gave detail to elucidate the changes in referral patterns. Our comparison was between mental health and A&E teams located in the same hospitals, thus mitigating any possible effects of geographical variations. We were able to compare trends in mental health presentations for the previous year, as well as comparing the impact of lockdown itself. We also compared these trends to numbers presenting to A&E departments.

Our study has limitations. We did not gather information about the numbers of patients presenting in crisis within primary care, secondary care community crisis services or accessing emergency psychiatric support other than via A&E departments or acute care centres (e.g. presenting to police or directly to crisis houses, etc.), so we cannot assess whether the reduction in presentations in these services was offset by these other services. London is a populous, diverse and urbanised capital city, and the results of this study may not be generalisable to other regions (also see Supplementary Appendix 1, section 6). In addition, there are a number of personal and policy factors that could change over the course of the pandemic, which could affect future psychiatry presentations, and we cannot account for or predict these. Our clinical record review was limited by the data recorded by clinicians, which reflected the opinion of the assessing clinicians and lacked detail such as patient educational level or socioeconomic status, which may have had an effect on trends in presentations. The review of over 2500 records required a team of clinicians, meaning potential variation in recording practice within the research team, but use of a standardised data collection form mitigated this. We were unable to assess diagnoses and other clinical characteristics of patient for the entire year because of a lack of resources to carry out such a detailed review of clinical notes. Additionally, we were only able to assess the impact of lockdown, as this occurred on a defined date. We were unable to assess the impact of the pandemic, which unfolded in a more gradual way, although the increase in psychiatric presentations in the month of February could be attributed to the World Health Organization's announcement of a public health pandemic. We also acknowledge that help-seeking for psychiatric symptoms is a complex process and likely to be influenced by social support, availability of services and networks, and illness-related symptoms, so further nuanced examination is necessary. Interpretation of the ITS is also limited by the opening of a mental health unit to divert psychiatric patients away from the main hospital A&E department, and assessing the impact of this on decisions to seek psychiatric help is beyond the scope of this study.

Our data are for a part of London with relatively well-funded mental health services that were able to restructure at short notice to meet changing demands post-lockdown. Despite this, there is a large change in terms of numbers of people presenting to mental health services and the severity of illness at presentation. If our results are representative of other psychiatric services, they suggest a very large burden of untreated mental illness at a time when services are stretched and there is less potential to restructure and increase provision. Reductions in psychiatric presentations may also be worse in areas where acute psychiatric services have been unable to create assessment centres separate from A&E services. Unlike A&E services, there is no national data collection on acute psychiatric presentations, which means tracking these changes in any meaningful way is difficult at present, and our data strongly suggest this type of national monitoring is necessary and useful for future planning.

Our findings show that lockdown resulted in a decrease in mental health presentations, but that those presenting had more severe symptoms, were more likely to be detained under the Mental Health Act and were more likely to be admitted to an in-patient unit after assessment. This suggests there is likely to be a future increase in pressure on psychiatric services, and we need robust monitoring in place, as well as an increase in funding and services, to be able to manage existing demand and account for a potential increase in demand because of COVID-19-related psychiatric morbidity. There also needs to be a plan in place for mental health provision for future waves of this pandemic and possible future pandemics.

## Data Availability

The data that support the findings of this study are available from the corresponding author, N.M., upon reasonable request.

## Supplementary material

For supplementary material accompanying this paper visit http://dx.doi.org/10.1192/bjo.2021.970. click here to view supplementary material

## References

[1] Zhu N, Zhang D, Wang W, Li X, Yang B, Song J, A novel coronavirus from patients with pneumonia in China. N Engl J Med 2020; 382: 727–33. 31978945 10.1056/NEJMoa2001017 PMC7092803

[2] Troyer EA, Kohn JN, Hong S. Are we facing a crashing wave of neuropsychiatric sequelae of COVID-19? Neuropsychiatric symptoms and potential immunologic mechanisms. Brain Behav Immun 2020; 87: 34–9. 32298803 10.1016/j.bbi.2020.04.027 PMC7152874

[3] Rogers JP, Chesney E, Oliver D, Pollak TA, McGuire P, Fusar-Poli P, Psychiatric and neuropsychiatric presentations associated with severe coronavirus infections: a systematic review and meta-analysis with comparison to the COVID-19 pandemic. Lancet Psychiatry 2020; 7(7): 611–27. 32437679 10.1016/S2215-0366(20)30203-0 PMC7234781

[4] Kelly BD. Coronavirus disease: challenges for psychiatry. Br J Psychiatry 2020; 217(1): 352–3. 32293555 10.1192/bjp.2020.86 PMC7205546

[5] Torales J, O'Higgins M, Castaldelli-Maia JM, Ventriglio A. The outbreak of COVID-19 coronavirus and its impact on global mental health. Int J Soc Psychiatry 2020; 66(4): 317–320. 32233719 10.1177/0020764020915212

[6] Luykx JJ, Vinkers CH, Tijdink JK. Psychiatry in times of the Coronavirus disease 2019 (COVID-19) pandemic: an imperative for psychiatrists to act now. JAMA Psychiatry 2020; 77(11): 1097–8. 32459359 10.1001/jamapsychiatry.2020.1225

[7] Rains LS, Johnson S, Barnett P, Steare T, Needle JJ, Carr S, Early impacts of the COVID-19 pandemic on mental health care and on people with mental health conditions: framework synthesis of international experiences and responses. Soc Psychiatry Psychiatr Epidemiol 2020; 56(1): 13–24. 32804258 10.1007/s00127-020-01924-7 PMC7429938

[8] Etheridge B, Spantig L. The Gender Gap in Mental Well-Being during the Covid-19 Outbreak: Evidence from the UK. Institute for Social and Economic Research, 2020 (https://www.iser.essex.ac.uk/research/publications/working-papers/iser/2020-08).

[9] Pierce M, Hope H, Ford T, Hatch S, Hotopf M, John A, Mental health before and during the COVID-19 pandemic: a longitudinal probability sample survey of the UK population. Lancet Psychiatry 2020; 7(10): 883–92. 32707037 10.1016/S2215-0366(20)30308-4 PMC7373389

[10] Keeter S. A Third Of Americans Experienced High Levels of Psychological Distress during the Coronavirus Outbreak. Pew Research Center, 2020 (https://www.pewresearch.org/fact-tank/2020/05/07/a-third-of-americans-experienced-high-levels-of-psychological-distress-during-the-coronavirus-outbreak/).

[11] Understanding America Study. Depression and Anxiety (14/06/20 ed). USC Center for Economic and Social Research, 2020 (https://covid19pulse.usc.edu/).

[12] Shi L, Lu Z-A, Que J-Y, Huang X-L, Liu L, Ran M-S, Prevalence of and risk factors associated with mental health symptoms among the general population in China during the Coronavirus disease 2019 pandemic. JAMA Network Open 2020; 3(7): e2014053-e. 32609353 10.1001/jamanetworkopen.2020.14053 PMC7330717

[13] Hoyer C, Ebert A, Szabo K, Platten M, Meyer-Lindenberg A, Kranaster L. Decreased utilization of mental health emergency service during the COVID-19 pandemic. Eur Arch Psychiatry Clin Neurosci [Epub ahead of print] 9 Jun 2020. Available from: 10.1007/s00406-020-01151-w. PMC7282463 32519205

[14] Surtees P, Kendell R. The hierarchy model of psychiatric symptomatology: an investigation based on present state examination ratings. Br J Psychiatry 1979; 135(5): 438–43. 540208 10.1192/bjp.135.5.438

[15] Department of Health and Social Care. Mental Health Act 1983: Code of Practice. The Stationery Office, 2015.

[16] Department for Constitutional Affairs. Mental Capacity Act 2005: Code of Practice. The Stationery Office, 2007.

[17] Linden A. Conducting interrupted time-series analysis for single-and multiple-group comparisons. Stata J 2015; 15(2): 480–500.

[18] Kontopantelis E, Doran T, Springate DA, Buchan I, Reeves D. Regression based quasi-experimental approach when randomisation is not an option: interrupted time series analysis. BMJ 2015; 350: h2750. 26058820 10.1136/bmj.h2750 PMC4460815

[19] Bernal JL, Cummins S, Gasparrini A. Interrupted time series regression for the evaluation of public health interventions: a tutorial. Int J Epidemiol 2017; 46(1): 348–55. 27283160 10.1093/ije/dyw098 PMC5407170

[20] Weisstein EW. Bonferroni Correction. Wolfram Mathworld, 2021 (https://mathworld.wolfram.com/BonferroniCorrection.html).

[21] Butler M, Delvi A, Mujic F, Broad S, Pauli L, Pollak TA, Reduced activity in an inpatient liaison psychiatry service during the first wave of the COVID-19 pandemic: comparison with 2019 data and characterization of the SARS-CoV-2 positive cohort. Frontiers Psychiatry 2021; 12: 619550. 10.3389/fpsyt.2021.619550 PMC7884445 33603687

[22] Chen S, Jones PB, Underwood BR, Moore A, Bullmore ET, Banerjee S, The early impact of COVID-19 on mental health and community physical health services and their patients’ mortality in Cambridgeshire and Peterborough, UK. J Psychiatr Res 2020; 131: 244–54. 33035957 10.1016/j.jpsychires.2020.09.020 PMC7508053

[23] Chen S, She R, Qin P, Kershenbaum A, Fernandez-Egea E, Nelder JR, The medium-term impact of COVID-19 lockdown on referrals to secondary care mental health services: a controlled interrupted time series study. Frontiers Psychiatry 2020; 11: 1307. 10.3389/fpsyt.2020.585915 PMC7726266 33324258

[24] Nishio A, Akazawa K, Shibuya F, Abe R, Nushida H, Ueno Y, Influence on the suicide rate two years after a devastating disaster: a report from the 1995 great Hanshin-Awaji earthquake. Psychiatry Clin Neurosci 2009; 63(2): 247–50. 19335397 10.1111/j.1440-1819.2009.01942.x

[25] Morali D, Jehel L, Paterniti S. The August 2003 heat wave in France: effects on psychiatric disorders and suicidal behavior. Presse Medicale (1983) 2007; 37(2 Part 1): 224–8. 18053678 10.1016/j.lpm.2007.06.025

[26] Gordon KH, Bresin K, Dombeck J, Routledge C, Wonderlich JA. The impact of the 2009 Red River flood on interpersonal risk factors for suicide. Crisis 2011; 32(1): 52–5. 21371971 10.1027/0227-5910/a000051

