# Supplementary material for: Acute mental health presentations before and during the COVID-19 pandemic
Source: BJPsych Open. 2021 Jul 16;7(4):e134. doi: 10.1192/bjo.2021.970 (PMC8314272; doi:10.1192/bjo.2021.970)
Supplement: Supplementary file 1 [file S2056472421009704sup001.docx]

**Contents of supplemental material**

[**1.** **Brief introduction** 1](#_Toc70701423)

[**2.** **ITS model for psychiatric presentations** 1](#_Toc70701424)

[**3.** **Alternative impact model for psychiatric presentations** 3](#_Toc70701425)

[**4.** **ITS model for A&E presentations** 4](#_Toc70701426)

[**5.** **Limitations of ITS models** 5](#_Toc70701427)

[**6.** **Comments on external validity** 5](#_Toc70701428)

[**7.** **Absolute numbers presenting to psychiatric services by presenting complaint.** 6](#_Toc70701429)

# **Brief introduction**

This supplementary material has been written to provide enough detail of the ITS analysis performed, to ensure its reproducibility. We summarise the two ITS analysis presented in the article, discuss on the assumptions and limitations of our models, and present complementary information on section 7.

# **ITS model for psychiatric presentations**

This is the hypothesised unadjusted segmented regression model for psychiatric presentations:

[Model 1]

$${log(Y}_{i})= \beta_{0}+\beta_{1}lockdown+\beta_{2}{time\_before}_{i}+\beta_{3}{time\_after}_{i}+ℇ_{i}$$

where *i* denotes the follow-up time (**in weeks**), $\beta_{1}$ is the intercept change that represents the immediate change at lockdown, $\beta_{2}$ is the before-lockdown slope and $\beta_{3}$ is the after-lockdown slope, and the outcome is expected to have a Poisson distribution. Since the latter assumption was not achieved, we included a Pearson X2-based dispersion parameter to adjust for overdispersion. We also included Fourier terms (cos(2 * degrees * pi / 180), sin(2 * degrees * pi / 180)) to each slope to adjust for seasonality and autocorrelation.

For Table 1 (in the article), the relative change (RC) is calculated as e^βk^. For example, for the variable “lockdown” in [Model 1], which represents the immediate change at lockdown, the RC = e^β1^ = e^-.2428^ = 0.7844. The next Table A shows the β estimates for both the unadjusted and adjusted models, accompanied with a graphical representation of the adjusted model (Figure A).

| Table A. Interrupted time series estimates for the effect of lockdown on psychiatric presentations (on a **weekly** basis) with **Model 1**. | | | | | | |
| --- | --- | --- | --- | --- | --- | --- |
| Model variable | Unadjusted* | | | Adjusted** | | |
|  | β | 95% CI | p | β | 95% CI | p |
| lockdown (β1) | -0.2136 | (-0.3346 to -0.0925) | 0.001 | -0.2428 | (-0.3936 to -0.0920) | 0.002 |
| time_before (β2) | -0.0014 | (-0.0033 to 0.0005) | 0.157 | -0.0016 | (-0.0036 to 0.0004) | 0.123 |
| time_after (β3) | 0.0060 | (0.0019 to 0.0101) | 0.004 | 0.0057 | (0.0004 to 0.0109) | 0.033 |
| sine_term_1 |  |  |  | -0.0586 | (-0.1043 to -0.0128) | 0.012 |
| sine_term_2 |  |  |  | 0.0086 | (-0.0291 to 0.0463) | 0.654 |
| cosine_term_1 |  |  |  | -0.0705 | (-0.1166 to -0.0244) | 0.003 |
| cosine_term_2 |  |  |  | -0.0093 | (-0.0476 to 0.0289) | 0.632 |
| constant (β0) | 5.2391 | (5.1693 to 5.3089) | <0.001 | 5.2601 | (5.1879 to 5.3323) | <0.001 |
| *(*) Adjusted for overdispersion only. The total number of time points is 104.* | | | | | | |
| *(**) Adjusted for overdispersion, autocorrelation and seasonality. The total number of time points is 104.* | | | | | | |

Figure A. Graphical depiction of the Interrupted time series estimates for the effect of lockdown on psychiatric presentations (on a **weekly** basis) with **Model 1**.

# **Alternative impact model for psychiatric presentations**

This is the sensitivity analysis referred to in the article. A feasible alternative model is one describing a gradual change after lockdown instead of an immediate change:

[Model 2]

$${log(Y}_{i})= \theta_{0}+\theta_{1}{time\_before}_{i}+\theta_{2}{time\_after}_{i}+ℇ_{i}$$

where *i* denotes the follow-up time (**in weeks**), $\theta_{1}$ is the before-lockdown slope and $\theta_{2}$ is the after-lockdown slope, and the outcome is expected to have a Poisson distribution. Since the latter assumption was not achieved, we included a Pearson X2-based dispersion parameter to adjust for overdispersion. We also included Fourier terms (cos(2 * degrees * pi / 180), sin(2 * degrees * pi / 180)) to each slope to adjust for seasonality and autocorrelation.

The Table B shows the estimates for the adjusted model:

| Table B. Interrupted time series estimates for the effect of lockdown on psychiatric presentations (on a **weekly** basis) with **Model 2**. | | | | | | |
| --- | --- | --- | --- | --- | --- | --- |
| Model variable | Unadjusted* | | | Adjusted** | | |
|  | θ | 95% CI | p | θ | 95% CI | p |
| time_before (θ1) | -0.0031 | (-0.0048 to 0.0014) | <0.001 | -0.0035 | (-0.0052 to 0.0018) | <0.001 |
| time_after (θ2) | 0.0009 | (-0.0021 to 0.0039) | 0.562 | -0.0013 | (-0.0043 to 0.0018) | 0.419 |
| sine_term_1 |  |  |  | -0.0166 | (-0.0558 to 0.0227) | 0.408 |
| sine_term_2 |  |  |  | 0.0149 | (-0.0431 to 0.0541) | 0.455 |
| cosine_term_1 |  |  |  | -0.1033 | (-0.1470 to -0.0597) | <0.001 |
| cosine_term_2 |  |  |  | -0.0056 | (-0.0456 to 0.0345) | 0.785 |
| constant (θ0) | 5.2757 | (5.2059 to 5.3454) | <0.001 | 5.3087 | (5.2403 to 5.3771) | <0.001 |
| *(*) Adjusted for overdispersion only. The total number of time points is 104.* | | | | | | |
| *(**) Adjusted for overdispersion, autocorrelation and seasonality. The total number of time points is 104.* | | | | | | |

[Model 2] is nested in [Model 1], so we made a comparison of the adjusted models based on a likelihood-ratio test:

Based on this comparison **we concluded [Model 1] has the best fit**; thus, it is the impact model reported in the article.

# **ITS model for A&E presentations**

This is the unadjusted segmented regression model for A&E presentations:

[Model 3]

$${log(Y}_{i})= \alpha_{0}+\alpha_{1}lockdown+\alpha_{2}{time\_before}_{i}+\alpha_{3}{time\_after}_{i}+ℇ_{i}$$

where *i* denotes the follow-up time (**in months**), $\alpha_{1}$ is the intercept change that represents the immediate change at lockdown, $\alpha_{2}$ is the before-lockdown slope and $\alpha_{3}$ is the after-lockdown slope, and the outcome is expected to have a Poisson distribution. Since the latter assumption was not achieved, we included a Pearson X2-based dispersion parameter to adjust for overdispersion. We also included Fourier terms (cos(2 * degrees * pi / 180), sin(2 * degrees * pi / 180)) to each slope to adjust for seasonality and autocorrelation.

For Table 1 (in the article), the relative change (RC) is calculated as e^αk^. For example, for the variable “lockdown” in [Model 3], which represents the immediate change at lockdown, the adjusted RC = e^α1^ = e^-.6514^ = 0.52. The next Table C shows the α estimates for both the unadjusted and adjusted models, accompanied with a graphical representation of the adjusted model (Figure B).

| Table C. Interrupted time series estimates for the effect of lockdown on A&E presentations (on a **monthly** basis) with **Model 3**. | | | | | | |
| --- | --- | --- | --- | --- | --- | --- |
| Model variable | Unadjusted* | | | Adjusted** | | |
|  | α | 95% CI | p | α | 95% CI | p |
| lockdown (α1) | -0.7210 | (-0.9279 to -0.5142) | <0.001 | -0.6514 | (-0.9041 to -0.3987) | <0.001 |
| time_before (α2) | 0.0044 | (-0.0072 to 0.0159) | 0.461 | 0.0025 | (-0.0096 to 0.0146) | 0.686 |
| time_after (α3) | 0.0524 | (0.0213 to 0.0834) | 0.001 | 0.0407 | (0.0025 to 0.0788) | 0.037 |
| sine_term_1 |  |  |  | 0.0212 | (-0.0525 to 0.0949) | 0.573 |
| sine_term_2 |  |  |  | 0.0227 | (-0.0360 to 0.0815) | 0.448 |
| cosine_term_1 |  |  |  | -0.0456 | (-0.1106 to 0.0195) | 0.170 |
| cosine_term_2 |  |  |  | 0.0517 | (-0.0081 to 0.1115) | 0.090 |
| constant (α0) | 10.7218 | (10.6157 to 10.8279) | <0.001 | 10.7362 | (10.6267 to 10.8457) | <0.001 |
| *(*) Adjusted for overdispersion only. The total number of time points is 24.* | | | | | | |
| *(**) Adjusted for overdispersion, autocorrelation and seasonality. The total number of time points is 24.* | | | | | | |

Figure B. Graphical depiction of the Interrupted time series estimates for the effect of lockdown on A&E presentations (on a **monthly** basis) with **Model 3**.

# **Limitations of ITS models**

**[Model 3]** has been fit on data that were available only on a **monthly basis**. This limited the number of time points modelled (24), affecting the statistical power. For that reason, we take the estimates from [Model 3] with caution. Additionally, any interpretation of change from [Model 3] should be made on a **monthly** basis, taking care that the event of interest (lockdown) occurred in a **specific day** of the month 15. Thus, the change reported in the next month is not strictly ‘abrupt’ in the way that each month-point reports all the presentations accumulated in that month. Having said that, [Model 3] should be interpreted -again - with caution.

**[Model 1]** is more robust because it was fit on a **weekly basis**. For example, the number of data points (104) allowed a better approximation of the seasonality patterns to control for. The abrupt change is also more realistic since the time windows are narrower (weeks instead of months).

Based on this explanation, any direct comparison between [Model 1] and [Model 3] is not possible. We tried to fit an alternative [Model 1] by generating monthly windows instead, but the procedure added too much noise and estimates were useless in practice. Since [Model 1] is the most robust as it is, we preferred to keep it and build our central conclusions on it.

# **Comments on external validity**

Our sample is not random; however, we believe it is broadly representative of London. This assumption is directly related to the main ITS impact model we defined in this study: an immediate change after lockdown followed by a gradual change (i.e., Models 1 and 3). Independently, Mansfiled et al^[[1]](#footnote-1)^ postulated and confirmed a similar ITS impact model for mental health services, analysing a bigger sample which is broadly representative of the UK. Their findings sustain our assumptions on the impact model itself, as well as on the direction of the change (immediate reduction followed by a slower recovery).

However, the same assumption cannot be made on the ITS effect sizes calculated from our sample. For that reason, we have been conservative in our analysis; for example, avoiding silent data from the lockdown initiation period in [Model 3] as Mansfield et al^1^ did properly in their own bigger and more granulated data – or not setting two slopes after lockdown in [Model 1] -what could fit better on our data-. Both tentative approaches would have produced bigger ITS effect sizes (e.g., higher immediate change after lockdown in Model 1) but mostly guided by the characteristics of our non-random sample. Having said that, and enlightened by Mansfield et al^1^ results, we believe our estimates are informative and conservative enough.

# **Absolute numbers presenting to psychiatric services by presenting complaint.**

| Presenting complaint | Psychosis | Violence/ aggression | Self-harm/Suicidal ideation | Delirium | Depressive symptoms | Anxiety | Intoxication |
| --- | --- | --- | --- | --- | --- | --- | --- |
| Before lockdown – total (per week) | 337 (28) | 80 (7) | 1033 (86) | 71 (6) | 134 (11) | 86 (7) | 66 (6) |
| After lockdown – total (per week) | 127 (21) | 27 (5) | 239 (40) | 38 (6) | 33 (4) | 22 (4) | 18 (3) |

1. Mansfield KE, Mathur R, Tazare J, Henderson AD, Mulick AR, Carreira H, Matthews AA, Bidulka P, Gayle A, Forbes H, Cook S. Indirect acute effects of the COVID-19 pandemic on physical and mental health in the UK: a population-based study. The Lancet Digital Health. 2021 Feb 18. https://doi.org/10.1016/S2589-7500(21)00017-0 [↑](#footnote-ref-1)
